# The roles of two extracellular loops in proton sensing and permeation in human Otop1 proton channel

**DOI:** 10.1038/s42003-022-04085-2

**Published:** 2022-10-20

**Authors:** Bin Li, Yan Wang, Alexis Castro, Courtney Ng, Zhifei Wang, Haroon Chaudhry, Zainab Agbaje, Gabriella A. Ulloa, Yong Yu

**Affiliations:** Department of Biological Sciences, St. John’s University, Queens, NY 11439 USA

**Keywords:** Membrane structure and assembly, Permeation and transport, Ion transport

## Abstract

Otopetrin (Otop) proteins were recently found to function as proton channels, with Otop1 revealed to be the sour taste receptor in mammals. Otop proteins contain twelve transmembrane segments (S1-S12) which are divided into structurally similar N and C domains. The mechanisms by which Otop channels sense extracellular protons to initiate gating and conduct protons once the channels are activated remains largely elusive. Here we show that two extracellular loops are playing key roles in human Otop1 channel function. We find that residue H229 in the S5-S6 loop is critical for proton sensing of Otop1. Further, our data reveal that the S11-12 loop is structurally and functionally essential for the Otop1 channel and that residue D570 in this loop regulates proton permeation into the pore formed by the C domain. This study sheds light on the molecular mechanism behind the structure and function of this newly identified ion channel family.

## Abstract (web summary)

Electrophysiology experiments, mutagenesis, and structural modelling provide insights into the structure and function of the sour taste receptor Otopetrin 1.

## Introduction

Humans, as well as most ﻿tested mammals, sense five basic tastes: sweet, bitter, sour, salty, and umami^1^. The taste system senses chemicals in food to help obtain nutrients and avoid noxious substances. Sour (or acid) taste senses acidic stimuli, which are often caused by noxious substances or toxins in the food, and trigger an aversive response to ensure they are not ingested^2^.

Compared to that of the other four tastes, the molecular mechanism of sour taste is less known and the primary receptor of sour taste was just discovered recently^3^. Tastes initially commence in subgroups of taste receptor cells (TRCs) on the tongue, when the membrane receptors in these TRCs are activated by soluble molecules^4,5^. TRCs responsible for sour taste were previously identified as a subgroup (Type III) of TRCs that specifically express the polycystic transient receptor potential (TRP) protein TRPP3 (also called polycystin-l)^6–9^. Previously, there was evidence suggesting that the ion channel complex formed between TRPP3 and the polycystic kidney disease (PKD) family protein PKD1L3 responds to acid and may serve as a sour taste receptor candidate^6,10–12^. However, this hypothesis was later challenged by animal studies showing that mice lacking TRPP3 or PKD1L3 still maintain a robust sour taste^13,14^. These results indicate that the PKD1L3/TRPP3 complex is not the primary sour taste receptor but leaves the question about its role in sour taste unanswered. Recently, based on differential RNA expression screen and electrophysiological recording, Tu et al. found that Otopetrin-1 (Otop1) is specifically expressed in type III TRCs and defined the Otopetrin proteins as proton-selective ion channels^15^. Following this discovery, animal studies further confirmed that Otop1 is indeed the primary sour taste receptor in mammals^16,17^. A recent study confirmed that Otop channels are directly gated by extracellular protons^18^. Interestingly, Otop1 was originally recognized to be essential for the development of otoconia that are involved in sensing gravity and acceleration^19–21^, which suggests a role of proton permeability in the vestibular system.

**Fig. 1:**
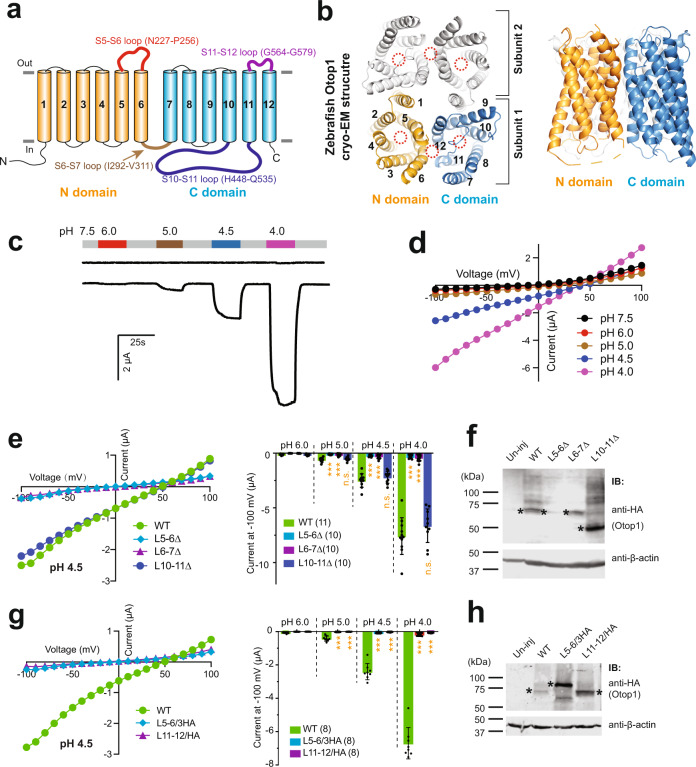
The extracellular S5–S6 loop and the S11–S12 loop are essential for Otop1 function. **a** The putative transmembrane topology of the human Otop1 channel, showing the N (transmembrane segments S1–S6) and C (S7–S12) domains, the loops connecting the TM segments, and the intracellular termini. Loops studied in this work are indicated. **b** The top (left) and side (right) views of the zebrafish Otop1 channel (PDB 6NF4)^23^. The numbers of the transmembrane segments are indicated on one of the subunits on the left. The three putative pores formed within the N and C domains and the intrasubunit interface between the two domains are indicated by dotted red circles. **c** Representative recording showing the currents of un-injected and human Otop1-injected oocytes at indicated pHs. Oocytes were clamped at −60 mV. **d** Representative current–voltage relationship (*I*–*V* curve) of human Otop1 channel at indicated pHs. **e**, **g** Left: the representative *I*–*V* curves of the indicated WT and mutant Otop1 channels. Right: the scatter plot and bar graph showing the currents of the indicated channels recorded at −100 mV at the indicated pHs. L5–6∆: S5–S6 loop deletion mutant; L6–7∆: S6–S7 loop deletion mutant; L10–11∆: S10–S11 loop deletion mutant; L5–6/3HA: the mutant with the S5–S6 loop replaced by three fused HA tags; L11–12/HA: the mutant with the S11–S12 loop replaced by an HA tag. Data in the bar graphs are presented as mean ± SD. Currents of the mutants were compared to that of the WT with Student’s *t*-test (***P < 0.001, n.s.: no significance). Oocyte numbers for scatter plots and bar graphs are indicated in parentheses. **f**, **h** Western blot images show the expression of the indicated Otop1 channels. Asterisks indicate the monomer bands of the WT and mutant proteins. Both L5–6/3HA and L11-12/HA gave stronger signals since they contain more than one copy of the HA tag. Mutant L5–6/3HA ran at a higher position on the gel for an unknown reason.

The cryo-EM structures of three Otop proteins, zebrafish Otop1, chicken Otop3, and *Xenopus* Otop3, were recently reported^22,23^. Since the sequences of Otop proteins do not share high similarity with any other ion channel family, the cryo-EM structures significantly improved our understanding of the structure and function of this new family of ion channels. All three Otop structures share common features. They are homodimers with each subunit containing 12 transmembrane segments (S1–S12) and intracellular N and C termini^22,23^ (Fig. 1a). The first six (S1–S6, the N domain) and the last six transmembrane segments (S7–S12, the C domain) of each Otop subunit form a barrel-like structure, respectively, which are surrounded by the transmembrane helices^22,23^ (Fig. 1b). Most interestingly, the two barrel-like structures formed by the N and C domains share a high structural similarity, despite their low sequence similarity.

Four possible proton-conducting cavities can be viewed from the cryo-EM structures^22–24^. The first is the central tunnel located between the two subunits (Fig. 1b). However, this tunnel was found to be completely blocked by bound lipid molecules and is most likely not involved in proton conduction^22,23^. Instead, molecular dynamic simulation and mutagenesis analysis indicated that the following three cavities may be involved in proton conductance: the putative pores in the center of the N and C barrels and the intrasubunit cavity which is located at the interface of the N and C domains^22,23^ (Fig. 1b). Although mutations in these putative pores were found to abolish the channel function^22,23^, how protons are conducted through these pores is still unknown. Additionally, the mechanism by which extracellular protons are sensed by Otop proteins to begin the channel’s gating process remains elusive. In this study, we explored the function of the loops linking transmembrane domains and found two extracellular loops that are essential for human Otop1 function. Key residues in these loops and their roles in proton sensing and permeation were identified. These results enhance our understanding of the molecular mechanism of the Otop1 channel’s function.

## Results

### The S5–S6 and the S11–S12 loops are essential for the function of human Otop1

As seen in many cases of structural determination, the majority of the long flexible loops connecting transmembrane segments are not resolved in the three published Otop cryo-EM structures^22,23^. Here we focused on the functional roles of four loops of significant length: the extracellular S5–S6 loop (named L5–6 which includes 30 residues from N227 to P256, predicted based on the cryo-EM structure of zebrafish Otop1^23^), the intracellular S6–S7 loop (L6–7, I292-V311, 20 residues), the intracellular S10–S11 loop (L10–11, H448-Q535, 88 residues), and the extracellular S11–S12 loop (L11–12, G564–G579, 16 residues) (Fig. 1a). Among them, only the shortest L11–12 was resolved in published cryo-EM structures of Otop proteins^22,23^.

We first confirmed the proton channel function of human Otop1 by expressing it in *Xenopus laevis* oocytes and recording it with the two-electrode voltage clamp (TEVC). In agreement with a previously reported study in HEK 293 cells^15^, expressing human Otop1 in oocytes gave rise to robust acid-induced currents (Fig. 1c, d). To further confirm that the current is carried by protons and to eliminate concerns that currents may come from background cation or anion channels in oocytes, we recorded the acid-induced currents in bath solutions containing different ions. First, we compared currents at pH 4 in bath solutions containing different cations: Na^+^, Li^+^, Cs^+^, and N-methyl-d-glucamine (NMDG^+^), as well as in a solution with Cl^−^ replaced by gluconate^−^. Results show that the currents and the reversal potentials we obtained in these solutions are almost identical to each other (Supplementary Fig. 1a). Second, the same acid-induced current was obtained in Otop1-expressing oocytes, but not in un-injected oocytes, in a solution containing organic salt tetraethylammonium (TEA) methanesulfonate and high concentrations of buffer (100 mM), which is similar to what was used in the previous study of the Hv1 proton channel^25^ (Supplementary Fig. 1b).

With these findings, we started testing the roles of the loops. In the first round of the experiments, we deleted 25 of 30 residues in L5–6, 13 of 20 residues in L6–7, and 78 of 88 residues in L10–11, respectively (see the “Methods” section for details). The recording results showed that shortening L5–6 or L6–7 nearly abolished the channel current, while shortening L10–11 did not affect channel activity (Fig. 1e). Based on these results, we first concluded that the longest L10–11 is not essential for channel activity in our experimental setting, although we cannot rule out its role in channel function regulation in vivo. Deletion of L6–7, which connects the N and the C domains (Fig. 1a), led to no change in protein expression, but rather, caused a loss of channel function (Fig. 1e, f). We believe shortening this loop will affect the formation of the proper intrasubunit interaction between the N and C domains, which has been shown to be critical for Otop channel function^22,23^. Deleting L5–6 also led to a nearly abolished channel function (Fig. 1e). Simultaneously, this mutation greatly reduced the channel expression (Fig. 1f). Thus, we were unable to make a solid conclusion on this loop based on this experiment.

Considering that the expression defect of the L5–6 deletion mutation may have been caused by a folding issue resulting from the shortened linker between S5 and S6, we further replaced this loop with three fused HA tags. This new linker has roughly the same length as the original L5–6. This mutant channel expressed well in oocytes (Fig. 1h), but still produced no current in recording (Fig. 1g). Thus, we believe that it is the specific residues in L5–6 that are important for channel function. Following the same strategy, we replaced the shortest L11–12 with one HA tag. Similarly, this channel had good expression but no channel activity (Fig. 1g, h). These results indicate that both L5–6 and L11–12 loops are essential for Otop1 channel function.

### H229 in L5–6 is critical for the function of the human Otop1 channel

**Fig. 2:**
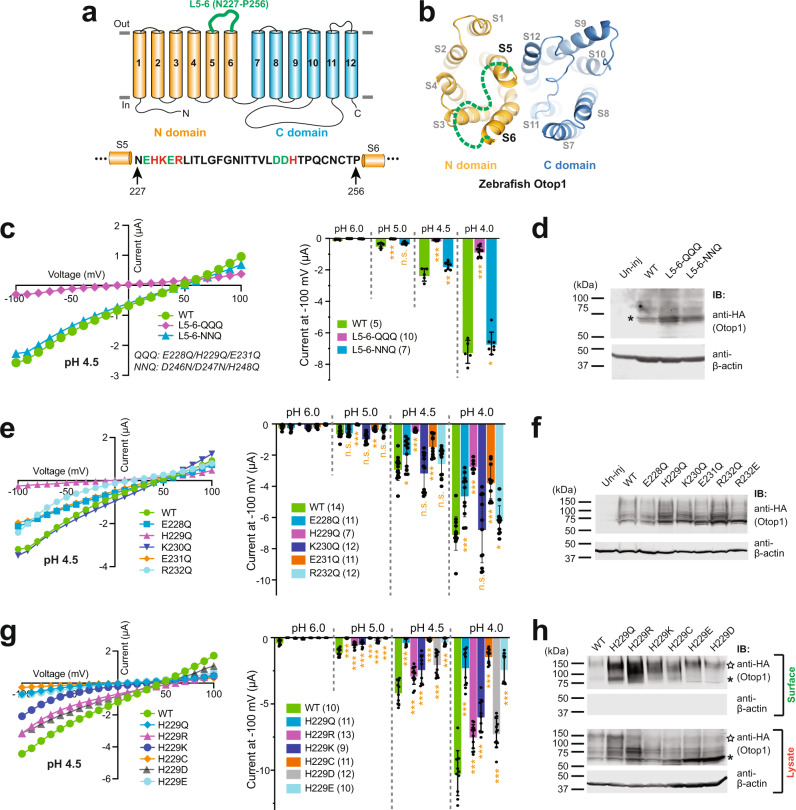
H229 in the L5–6 is crucial for the function of the Otop1 channel. **a** Schematic illustration shows the locations of the L5–6 in the topology structure of Otop1. The amino acid sequence of the loop is provided at the bottom and the negatively and positively charged residues are in green and red, respectively. **b** The cryo-EM structure of the zebrafish Otop1 (PDB 6NF4)^23^. The S5–S6 loop is not resolved in the structure and the green dotted line is utilized to label its relative position in the N domain. **c**, **e**, **g** Left: the representative *I*–*V* curves of the indicated WT and mutant Otop1 channels. Right: the scatter plot and bar graph showing the currents of the indicated channels recorded at −100 mV at the indicated pHs. Data in the bar graphs are presented as mean ± SD. Currents of the mutants were compared to that of the WT with Student’s *t*-test (*P < 0.05, **P < 0.01, ***P < 0.001, n.s.: no significance). Oocyte numbers for scatter plots and bar graphs are indicated in parentheses. **d**, **f** Western blot showing the expression of the indicated WT and mutant channels. **h** Western blot showing the overall expression (in lysate samples) and surface expression of indicated WT and mutant channels. Surface proteins were purified by surface biotinylation. The asterisks and stars show the monomer and dimer bands of Otop1, respectively.

Our results above show that L5–6 and L11–12 are essential for the Otop1 channel function. We next sought to determine the key amino acids in these two loops. L11–12 is resolved in all three published cryo-EM structures and is located at the top opening of the putative C pore^22,23^. By contrast, L5–6 is not resolved in any cryo-EM structure, suggesting a flexible structure of this loop. Based on the primary sequence of Otop1, this loop may be located close to the top opening of the putative N pore (Fig. 2a, b). Meanwhile, the loop is long enough to reach the top opening of the C pore as well as that of the intrasubunit interface. Since Otop channels sense protons and conduct them through the putative pores, we hypothesize that the charged amino acids in both loops may influence proton sensing and conductance. Thus, we shifted our focus to these charged amino acids in L5–6 and L11–12.

In the 30-residues long L5–6, there are two clusters of consecutive charged amino acids: E228-R232 and D246-H248 (Fig. 2a). In the first round of experiments, we neutralized all negatively charged amino acids and the two histidine residues in the two charge clusters by generating two mutants, each carrying three mutated residues simultaneously: E228Q/H229Q/E231Q (named “L5–6-QQQ”) and D246N/D247N/H248Q (named “L5–6-NNQ”). Our results show that the current of L5–6-QQQ was very small compared to that of the wild-type (WT) channel, despite its good expression (Fig. 2c, d). By contrast, the mutant L5–6-NNQ only caused a very mild reduction in channel activity (Fig. 2c, d). These results suggest that the first, but not the second, charge cluster is critical for channel function.

We then neutralized each charged residue in the first charge cluster, including the two positively charged amino acids K230 and R232, to non-charged glutamine (Q). The results demonstrate that, aside from K230Q, all the other mutations (E228Q, H229Q, E231Q, R232Q) cause some degree of reduction of the current amplitude at tested acidic pHs, despite the expression level being comparable to or greater than the WT (Fig. 2e, f). The greatest channel activity reduction was seen in the H229Q mutant, which caused a nearly complete loss of proton current at pH 5 and 4.5 and about 60% reduction of current at pH 4 (Fig. 2e). This result suggests that H229 has an essential role in channel function. Focusing on this residue, we mutated it into residues with different charge properties. In addition to the uncharged Q, we also mutated it to the positively charged arginine (R) and lysine (K), negatively charged aspartic acid (D) and glutamic acid (E), and uncharged cysteine (C). The recording data show that all mutations caused some degree of reduction in the channel current amplitude (Fig. 2g). Among all the mutations, H229Q, H229C, and H229E gave rise to almost no current at pH 5 and pH 4.5 and dramatically reduced currents at pH 4, compared to the WT channel (Fig. 2g). Mutants with not only the positively charged H229R and H229K but also the negatively charged H229D at this position all have relatively good channel activities (Fig. 2g). To confirm that the reduced function of the mutants was not caused by issues in protein trafficking, we detected the plasma membrane expression of all H229 mutants with the surface biotinylation method. The results demonstrate good surface expression of all mutants (Fig. 2h). It is worth pointing out that in our experiments, the appearance of the dimer or higher oligomer bands of Otop1 on SDS–PAGE gel appears to be a random event, which may be affected by sample preparation. However, we noticed that the dimer band is always present and is heavier than the monomer band in all surface samples (Fig. 2h).

### H229 in L5–6 is essential for proton sensing of the human Otop1 channel

**Fig. 3:**
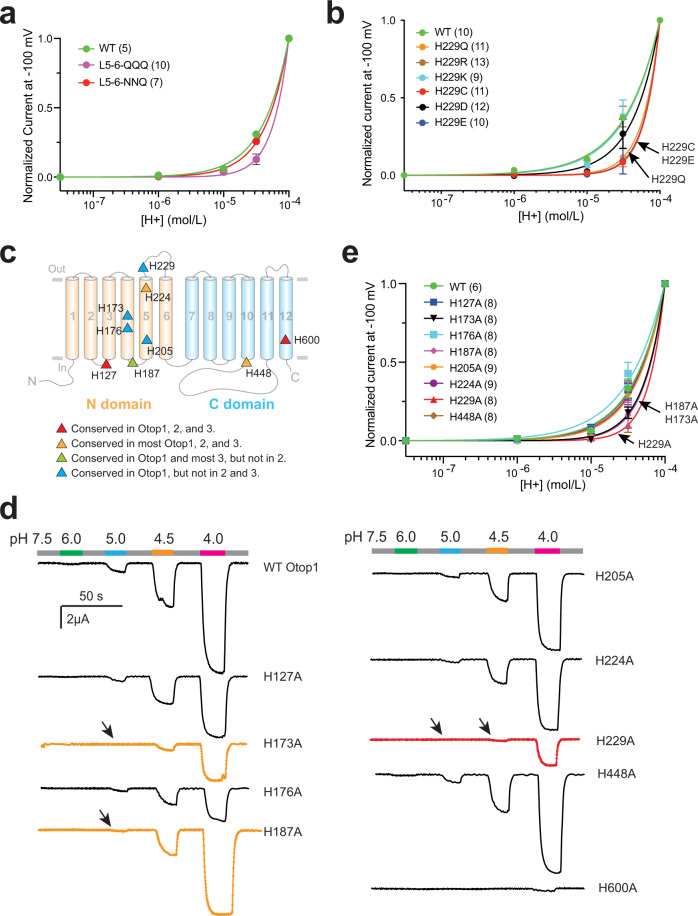
H229 in L5–6 is a key residue for the proton sensing of the human Otop1 channel. **a**, **b**, **e** Proton dose–response curves showing the acid sensitivity comparison between the WT and the indicated mutant channels. The currents of every oocyte at other pHs were normalized to the current at pH 4 at −100 mV. The data were fitted by nonlinear regression with variable slope. We were unable to calculate an accurate EC_50_ of these channels. Since it is not possible to separate the proton sensing and proton conductance in this case, theoretically, we are not able to obtain the saturated proton concentration. **c** Predicted positions of indicated histidine residues in the topology structure of human Otop1. The residues are colored based on their conservation in Otop proteins as indicated at the bottom. **d** Gap-free recording shows the currents of indicated histidine mutants at different pHs. The oocytes were held at −60 mV. The currents of H229A, H173A, and H187A, the three mutants that have altered proton sensitivity, are in red or orange. Arrows indicated the missing responses to protons at the indicated pHs.

Otop channels are gated by the increase of proton concentration at the extracellular side^18^. Thus, a mechanism sensing extracellular protons must be present. Because histidine is partially protonated at pH 7.0 and fully protonated at low pH, it has been found to play a role in sensing pH in various proteins, including proton-gated ion channels^26–29^. Is H229 involved in proton sensing by the Otop1 channel? The first evidence of its involvement arose from the L5–6-QQQ mutant channel, which contains the H229Q mutation. After plotting the currents to proton concentrations to generate the dose–response relationship, we noticed that the L5–6-QQQ mutant showed a less sensitive response to proton increase compared to the WT channel (Fig. 3a). It is worth mentioning that, in this case, since separating proton sensing and proton conductance is impossible and the proton current does not reach saturation in the tested pH range, we were unable to acquire the protons’ EC_50_ of either the WT or the L5–6-QQQ channel. Thus, we used the currents at higher pHs relative to the currents at pH 4 as a measure of pH sensitivity. Next, we generated the dose–response relationship of the H229 mutants. The results show that the H229R and H229K mutants have similar proton sensitivity as the WT channel, while H229D has slightly reduced proton sensitivity (Figs. 3b and  2g). Meanwhile, Otop1 proton sensitivity is greatly reduced by the other three H229 mutations, H229Q, H229C, and H229E (Figs. 3b and 2g). These results suggest that a positive charge is favorable but not required at the H229 position for Otop1 to sense protons.

To further investigate the proton sensing of the Otop1 channel, we mutated nine most conserved histidine residues, including H229, in the human Otop1 channel to alanine individually (Fig. 3c). The conservation of these histidine residues is indicated in Fig. 3c based on the alignment of the protein sequences of Otop1, 2, and 3 in zebrafish, *Xenopus*, snake, chicken, mouse, and human. In our testing, one of the mutants, H600A, which is located at the putative intrasubunit interface pore, completely abolished channel current (Fig. 3d, Supplementary Fig. 2). This result is consistent with a previous report of a corresponding mutation in zebrafish Otop1 and *Xenopus* Otop3^22,23^. To further evaluate the role of H600, we mutated it to negatively charged aspartic acid (D) and glutamic acid (E), the positively charged lysine (K) and asparagine (N), as well as the non-charged glutamine (Q) and arginine (R). All these mutants are functional to some degree and showed similar proton sensitivity as the WT Otop1 channel (Supplementary Fig. 3). Thus, H600 is not involved in sensing protons. Five other mutants, H127A, H176A, H205A, H224A, and H448A also exhibit similar proton sensitivity as the WT Otop1 (Fig. 3d, e, Supplementary Fig. 2). Meanwhile, the other three mutations, H173A, H187A, and H229A caused the greatest decrease in proton sensitivity (Fig. 3d, e, Supplementary Fig. 2). Although these results suggest the involvement of all these three histidine residues in determining the proton sensitivity of the Otop1 channel, we believe that H229 may be more critical in sensing extracellular proton since both H173 and H187 should not be accessible directly from extracellular side based on the reported cryo-EM structures (see the “Discussion” section).

### L11–12 is structurally and functionally essential for the Otop1 channel

**Fig. 4:**
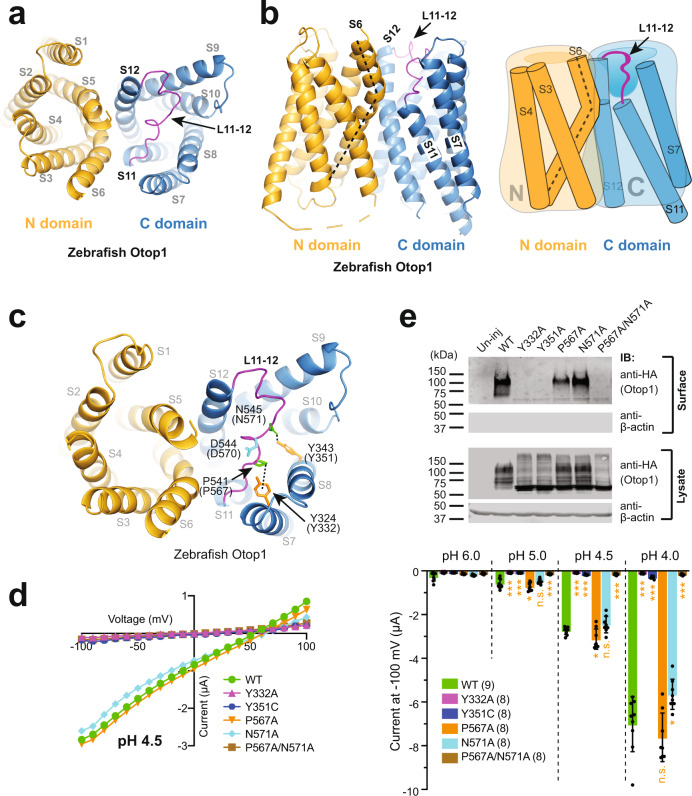
L11–12 is structurally and functionally essential for the Otop1 channel. **a** Top view of the cryo-EM structure of the zebrafish Otop1 (PDB 6NF4)^23^, showing that L11–12, which is in purple, crosses the center of the C pore. **b** Side view of the cryo-EM structure of the zebrafish Otop1 (left) and a cartoon (right) show the gap between S7 and S12 in the top 1/3 portion of the C barrel caused by the relatively lower position of S11 in the transmembrane part of the Otop1 structure. L11–12 extends upwards and crosses the pore to reach S12. S6 is bent (indicated by dashed lines) to further leave the gap open for access. **c** Top view of the cryo-EM structure of the zebrafish Otop1 to show the two very conserved tight interactions (shown in the black dotted line) that anchor L11–12 in the right position. Side chains of the four residues involved in the interactions, as well as D544 (D570 in human), are shown as sticks. Residue numbers are indicated in both zebrafish and human (in parenthesis) sequences. **d** Left: the representative *I*–*V* curves of the indicated WT and mutant human Otop1 channels. Right: the scatter plot and bar graph showing the currents of the indicated channels recorded at −100 mV at the indicated pHs. Data in the bar graphs are presented as mean ± SD. Currents of the mutants were compared to that of the WT with Student’s *t*-test (*P < 0.05, ***P < 0.001, n.s.: no significance). Oocyte numbers for scatter plots and bar graphs are indicated in parentheses. **e** Western blot showing the overall expression (in lysate samples) and surface expression of indicated WT and mutant channels. The surface proteins were purified by surface biotinylation.

The last two transmembrane segments S11 and S12, as well as the L11–12 loop which connects them, possess the most conserved sequences in all three Otop family members across different species. In the cryo-EM structure of the zebrafish Otop1 channel, L11–12 spins across the top opening of the putative pore of the C domain (Fig. 4a)^23^. Interestingly, compared to the other transmembrane segments, the S11 helix stays at a lower position in the transmembrane structure of Otop1 and leaves a gap in the top portion of the C barrel between S7 and S12 (Fig. 4b)^23^. The unique position of S11 makes it appear visually as the “shortest board of the wooden barrel” in the C domain (Fig. 4b). The result of this configuration is that L11–12 begins at the top 1/3 position of the barrel, where it extends upwards and crosses the pore to reach S12. Thus, this loop is actually located inside the C pore, partially patching the gap formed above S11 and covering the top opening of the pore. Meanwhile, the bottom half of S6 in the N-domain is tilted toward the C-domain in the cryo-EM structures of zebrafish Otop1^23^. If S6 is a straight helix, it will extend into the gap on top of S11 to fill the S7/S12 gap (Fig. 4b). However, S6 bends at the level of the top end of S11, which further ensures that the S7/S12 gap is open (Fig. 4b). This structural feature is also conserved among three available Otop1 and Otop3 structures^22,23^. Thus, protons may also enter the C pore from this S7/S12 gap aside from the top opening. In both ways, L11–12 will likely play a role in regulating proton permeation.

Further analysis of the position of L11–12 in the zebrafish Otop1 cryo-EM structure^23^ led us to recognize two highly conserved interactions that anchor L11–12 to its current position in the structure. The first is the CH/π interaction between P541 (P567 in human Otop1) in L11–12 and Y324 (Y332 in human Otop1) in S7. The second interaction is a hydrogen bond that hooks N545 (N571 in human Otop1) in L11–12 and Y343 (Y351 in human Otop1) in S8 (Fig. 4c). All residues are highly conserved among Otop 1, 2, and 3 proteins in distinct species, and both interactions are observed in all three published cryo-EM structures of Otop1 and Otop3 proteins^22,23^. These two tight interactions anchor L11-12 at the center of the top opening of the C domain (Fig. 4c). Apart from maintaining the structure of L11–12, they may also play a key role in protein assembly by stabilizing the top portions of S7 and S8. To test how these interactions contributed to the proper structure and function of Otop1, we created the single amino-acid mutations Y332A, Y351C, P567A, and N571A, and the double amino-acid mutation P567A/N571A. The results show that mutants on S7 and S8, Y332A and Y351C, completely abolish the Otop1 channel function (Fig. 4d). Single mutations on the S11–S12 loop, P567A, and N571A, have mild effects on channel activity. However, mutating both residues simultaneously (P567A/N571A) abolished channel function (Fig. 4d). Further surface expression analysis shows that although the overall expression is fine, Y332A, Y351C, and P567A/N571A are unable to traffic to the plasma membrane, resulting in abolished channel current (Fig. 4e). By disrupting the interactions anchoring L11–12, these mutations should have led to flexibility in the structure of L11–12 as well as the positions of S7 and S8, which appear to be essential for protein folding and trafficking. At the same time, single point mutations P567A and N571A did not affect expression and trafficking (Fig. 4e). We assume that mutating either one of these two residues is not enough to break the interaction between L11–12 and two transmembrane domains S7 and S8. Together, these data suggest that L11–12 is structurally critical to the Otop1 protein.

### D570 in L11–12 is crucial for the function of the Otop1 channel

**Fig. 5:**
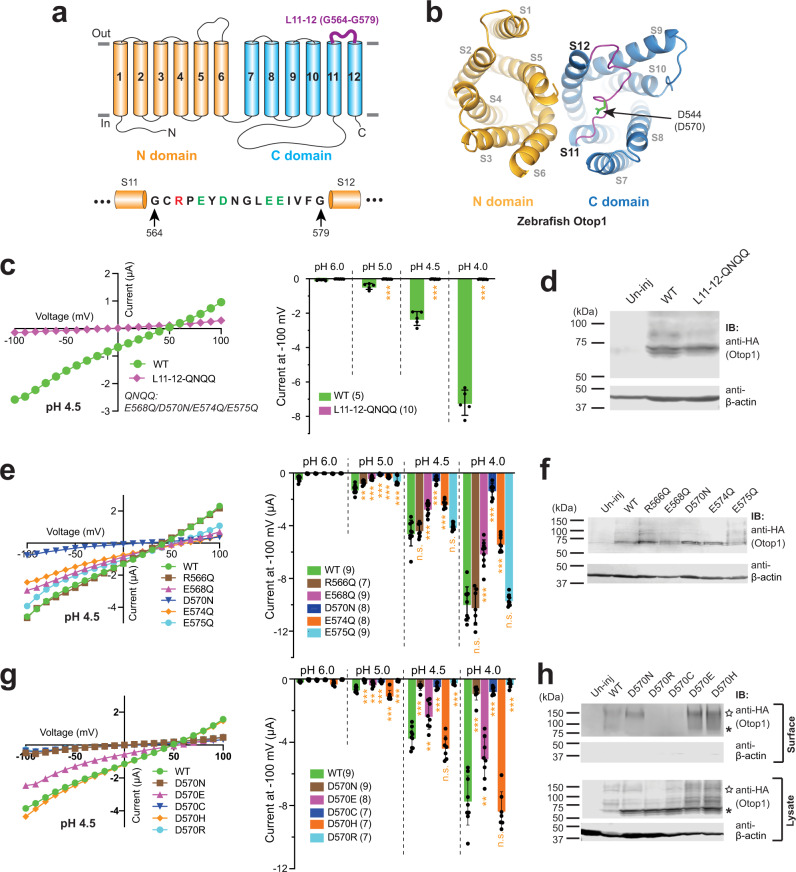
D570 in L11–12 is critical for the function of the Otop1 channel. **a** Schematic illustration shows the locations of the L11–12 in the topology structure of Otop1. The amino acid sequence of the loop is presented at the bottom and the negatively and positively charged residues are in green and red, respectively. **b** The cryo-EM structure of the zebrafish Otop1 (PDB 6NF4)^23^. The S11–S12 loop, which is in purple, is shown from the top views. The side chain of D544 (corresponding to D570 in human Otop1) is shown as green sticks. **c**, **e**, and **g** Left: the representative *I*–*V* curves of the indicated WT and mutant Otop1 channels. Right: the scatter plot and bar graph showing the currents of the indicated channels recorded at −100 mV at the indicated pHs. Data in the bar graphs are presented as mean ± SD. Currents of the mutants were compared to that of the WT with Student’s *t*-test (*P < 0.05, ***P < 0.001, n.s.: no significance). Oocyte numbers for scatter plots and bar graphs are indicated in parentheses. Currents in bar graphs are normalized to the mean of the WT currents at pH 4 recorded from the same batch of oocytes. **d**, **f** Western blot showing the expression of indicated WT and mutants. **h** Western blot showing the overall expression (in lysate samples) and surface expression of indicated WT and mutant channels. The surface proteins were purified by surface biotinylation. The asterisks and stars indicate the monomer and dimer bands of Otop1, respectively.

We next delved deeper into investigating the roles of charged residues in L11–12. This 16-amino-acid long loop incorporates a positively charged R566 and four negatively charged residues (E568, D570, E574, and E575) (Fig. 5a, b). We first neutralized all four negatively charged residues by generating a mutant containing E568Q/D570N/E574Q/E575Q simultaneously (named “L11–12-QNQQ”). Although the mutant channel was expressed well in oocytes, it resulted in the complete loss of proton channel activity (Fig. 5c, d), indicating at least one of these negatively charged residues is important for channel function.

We then generated separate mutants that neutralized each charged residue, including R566. We found that R566Q and E575Q did not change the size of the current, while mutations E568Q, D570N, and E574Q, all diminished the current amplitude to some degree, despite having an expression level comparable to or higher than that of WT (Fig. 5e, f). These results reveal that these negative charges on the S11–S12 loop are essential for channel function. Comparatively, D570N has the most drastically reduced current than any other mutant, indicating it has a critical role in channel function (Fig. 5e). D570 is highly conserved in Otop1 proteins. In the cryo-EM structure of zebrafish Otop1^23^, D570 is located at the center of the S11–S12 loop (Fig. 5b). Similar to the H229 experiments described above, we further mutated D570 to different residues. In addition to uncharged asparagine (N), we also changed it to negatively charged glutamic acid (E), uncharged cysteine (C), and positively charged arginine (R) and histidine (H). The channel activity and surface expression of all mutants were tested. The results show successful surface expression of the D570N channel, confirming that the reduced current amplitude in this mutant was not caused by a defect in channel trafficking (Fig. 5h). Additionally, D570R and D570C mutants gave rise to very small currents (Fig. 5g). However, both mutations also dramatically reduced the surface expression of the channels (Fig. 5h), although we observed a significant surface signal of D570C after loading more samples in the gel (Supplementary Fig. 4). Since the two mutants have weaker surface expression, we were unable to conclude the effects of these mutations on channel function. D570E has good surface expression and a smaller, but still robust, current than the WT channel, suggesting that a negative charge at this position is sufficient to keep channel activity (Fig. 5g, h). Indeed, a glutamic acid replaces the aspartic acid at this position in the Otop3 proteins of most species. Interestingly, when D570 was mutated to H, the channel activity did not change (Fig. 5g, h). Although this result indicates that a negative charge is not necessary for channel activity at this position, we could not rule out the possibility that the D570H mutation causes other conformational changes in the C pore, which helps preserve channel activity (see the “Discussion” section). Overall, our results suggest an essential role of D570 in the Otop1 function.

### D570 is in the proton-conducting pathway

To further examine the roles of H229 and D570 in Otop1 channel function, we utilized the substituted cysteine accessibility method (SCAM) to explore whether H229 and D570 are in the ion-conducting pathways. SCAM is a widely applied method in mapping channel pore-lining residues and detecting dynamic structural changes of ion channels at different functional states^30^. To perform SCAM, one mutates the target residues to cysteine and tests its accessibility to react with small, charged, and sulfhydryl-specific molecules such as the methanethiosulfonate (MTS) reagents. This reaction leads to the reversible chemical modification of the cysteine and may contribute to an alteration of the channel function, especially when the mutated cysteine is located in the ion-conducting pathway^30^.

**Fig. 6:**
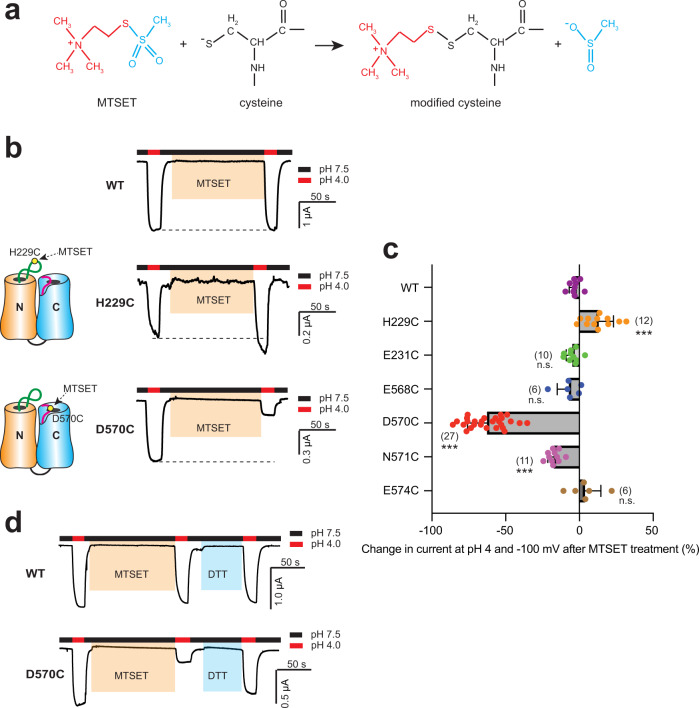
SCAM confirms the roles of H229 and D570 in proton conductance. **a** Schematic showing the modification of MTSET on cysteine. **b** Gap-free recording showing the currents of WT and mutant Otop1 channels at pH 4 before and after 2-min treatment of 1 mM MTSET. Oocytes were clamped at −60 mV. Dashed lines indicate the current sizes before MTSET treatment. The cartoons on the left demonstrate the reaction of MTSET with the substituted cysteine residues. **c** The scatter plot and bar graph showing the effect of MTSET treatment on indicated WT and mutant Otop1 channels. Currents at pH 4 after 1 mM MTSET treatment for 2 min are compared with that before treatment. Data in the bar graphs are presented as mean ± SD. Effects on the mutants were compared to that on the WT with Student’s *t*-test (***P < 0.001, n.s.: no significance). The numbers of oocytes in each group are indicated in parentheses. **d** Gap-free recording showing that the application of DTT (50 mM) for 1 min can rescue the effect of MTSET treatment on the D570 mutant. Meanwhile, the WT channel was not affected by DTT treatment.

We used the positively charged 2-(Trimethylammonium)ethyl methanethiosulfonate (MTSET) to test Otop1 channels with mutations H229C, D570C, and several other cysteine mutations located nearby. The reaction between MTSET and free cysteine results in a bulky and positively charged group on cysteine^30^ (Fig. 6a). The covalent medication by MTS can be reversed by applying reducing reagents such as 1,4-dithiothreitol (DTT). To obtain a negative control, we first tested the effect of MTSET treatment on the WT Otop1 channel and found that after a 2-min treatment with 1 mM MTSET at pH 7.5, no change in current amplitude was observed at pH 4 (Fig. 6b, c). Thus, MTSET treatment did not lead to a detectable functional change in the WT channel. Next, we found that MTSET treatment led to a slight but significant (~12%) increase in the H229C current, as well as a dramatic decrease (~62%) in the D570C current (Fig. 6b, c). The current of D570C can be rescued by applying 50 mM DTT for 1 min (Fig. 6d), confirming that MTSET treatment blocks the channel by modifying D570C. These results suggest that D570 most likely is located right in the proton-conducting pathway, supporting the critical role of this residue. To further explore the modification of D570C, in another experiment, we treated the Opto1-D570C mutant with three MTS reagents with the same positive charge but different sizes: MTSEA, MTSET, and MTS-PtrEA (Supplementary Fig. 5). The results show that all three of them lead to significant (≥60%) inhibition to the channel current at pH 4, suggesting even the MTSEA modification is large enough to block the proton conductance (Supplementary Fig. 5). We also noticed that treating with bigger MTS reagents tends to cause greater inhibition of the Otop1-D570C current (Supplementary Fig. 5), consistent with the hypothesis that D570C is in the proton-conducting pathway. It is worth mentioning that we cannot rule out the possibility that MTS modification of D570C inhibits the channel activity via an allosteric effect. In contrast, the results of H229C suggest that H229 might not be directly located in the proton-conducting pathway, and thus modification by MTSET does not block the channel. We hypothesize that the slightly increased channel activity of H229C after MTSET modification was caused either by conformational changes that occurred following MTSET treatment, or that the modification at this position led to a higher sensitivity of the channel to protons. The second hypothesis is consistent with H229 playing a key role in proton sensitivity (Fig. 3e).

To confirm the significant roles of these two residues, we also mutated and tested the following residues surrounding H229 and D570: E231, R232, E568, N571, and E574. One of the mutants, R232C, abolished channel current (Supplementary Fig. 6). Among the other mutants, MTSET treatment caused a 17% reduction of channel current in N571C, the residue next to D570 (Fig. 6c, Supplementary Fig. 6). The effects of MTSET treatment were mild in the other mutants (Fig. 6c, Supplementary Fig. 6). These results further indicate that, among the tested residues, D570 is the most critical one in proton conductance in these two regions.

## Discussion

As a newly identified ion channel family, the structures of the Otop channels are unlike other known ion channels. Their unique structures drive us to investigate the molecular mechanism of the channel’s function and regulation.

Previously, histidine had been found to play the role of pH sensors in pH-regulated ion channels, such as the Kir potassium channel^29^, the HCN2 channel^26^, and the influenza M2 proton channel^27^. In most cases, histidine is further protonated at a low pH and the increased positive charge drives conformational changes beyond the residue. In this study, we identified three histidine residues, H173, H187, and H229, that are potentially involved in human Otop1 proton sensing (Fig. 3). H229 is located in L5–6, which we have found to be critical for the Otop1 channel function (Fig. 1). Since this residue can be easily accessed from the extracellular side, it is reasonable for it to play a role in directly sensing extracellular protons. This effect is specific since mutating the non-conserved H83, a residue located on the extracellularly accessible S1–S2 loop of human Otop1 when it is mapped to the zebrafish Otop1 cryo-EM structure, did not change the proton sensing of human Otop1 (Supplementary Fig. 7). Compared to H229, the manner in which H173 and H187 play their roles in proton sensing is not obvious, since both are not directly accessible from the extracellular side. Mapping to the zebrafish Otop1 structure, H173 is located in the middle of S4 with its side chain facing towards the center of the N pore, while H187 is located at the intracellular loop between S4 and S5 (Fig. 3c)^23^. Thus, it is possible that mutation of these two residues leads to allosteric effects which change proton sensitivity. The role of H229 in proton sensing can be specific to the Otop1 channel since although the residue is very conserved in Otop1 channels, it is not in Otop2 and 3. Indeed, the proton gating properties of the three Otop channels have been shown to differ from each other^15,18^. Simultaneously, since H229R and H229K mutants retain good proton sensitivity (Figs. 2g and  3b), it seems that the role that H229 is playing in Otop1 proton sensing may not be direct via protonation-induced conformational changes. Meanwhile, since H229 mutants can still function as proton-activated proton channels, we hypothesize that Otop1 has a proton-sensing mechanism beyond the role of H229 found in this study. During the revision of this manuscript, Teng et al. reported their new findings on the gating of Otop channels. By swapping the extracellular loops between Otop2 and Otop3, they concluded that some of these loops, including L5–6, where H229 is in, are involved in the proton gating and that the gating apparatus is distributed across multiple loops^18^. These results are consistent with our conclusion that although H229 is a key residue, it most likely is only a part of the proton sensing apparatus. It is worth noting that in two recent studies, a histidine and a group of acidic amino acids were proposed, respectively, to be the proton sensor in a proton-activated chloride channel formed by TMEM206 proteins^28,31^. We did not notice changes in the proton sensing after mutating some acidic residues in the current study, although we could not rule out the possibility that other acidic residues are involved in the proton sensing of Otop1.

The unique location of the L11–12 in the C domain (Fig. 5a, b) puts it in a perfect position to regulate proton permeation into the C pore. Due to the lower position of S11 and the bend of S6, an open gap forms in the top 1/3 portion of the C barrel between S7 and S12, which is patched by L11–12. There are four negatively charged residues in the S11–S12 loop, which greatly contribute to the negative electric surface potential at the top of the C pore. This negative potential can be crucial for attracting protons into the pore. Indeed, our results demonstrate that these residues are important for channel function, with D570 neutralization notably leading to the most dramatic effect (Fig. 5e). Further investigation of this residue shows that a negative charge at this position is favored for channel trafficking and function (Fig. 5g, h). Interestingly, mutating D570 to histidine preserves the channel activity, despite the charge becoming positive (Fig. 5g). Considering the property of histidine residue at low pH, we hypothesize that the D570H mutation itself or the protonation of D570H at low pH causes a conformational change in the C pore, which compensates for the effect caused by the loss of the negative charge by an unknown mechanism.

**Fig. 7:**
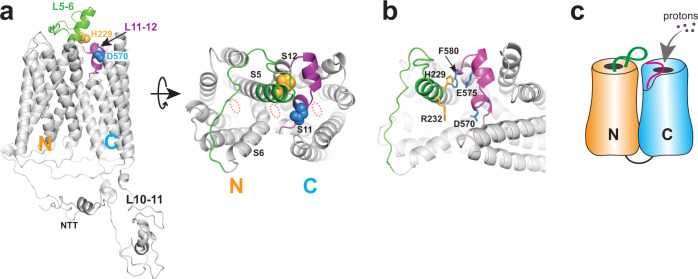
A structural model of the human Otop1 channel. The structure was built with the deep learning method RoseTTAFold^32^. **a** The side (left) and top (right) views of the model, show the positions of the L5–6 (green) and L11–S12 (purple). The first 1/3 of the putative L5–6 was predicted to be an extension of the S5 helix. Side chains of H229 (orange) and D570 (blue) are shown as spheres. The three putative pores are indicated by dotted orange ellipses. NTT N-terminal tail. **b** An enlarged view to show the predicted interactions between H229 and F580, and between H229 and E575. R232 is also shown here. It is predicted to point to the pore formed at the intracellular interface. **c** Cartoon showing the possible initiation of proton permeation at the extracellular side of the C pore.

To gain more insight into the roles of the S5–S6 and S11–S12 loops in human Otop1, we generated a structural model of this protein with the recently published deep-learning method RoseTTAFold, which has been proven to be able to provide accurate predictions of protein structures in many cases^32^. In this model, the S11–S12 loop, resembling its position in the cryo-EM structures of other Otop channels, spins across the top of the C pore directly (Fig. 7a). D570 is located right above the C pore and is open to the extracellular side in this model. In our SCAM experiments, MTS reagents can access and modify D570C at pH 7.5. These results also suggest that D570 is accessible from the extracellular side even when the channel is closed. If protons enter the C pore from the top opening, having a negatively charged residue at this site will most likely regulate proton conductance. This is also consistent with our results demonstrating that the addition of a bulky side chain on D570C with MTS treatment dramatically blocks the current amplitude (Fig. 6). Thus, our data strongly suggest that protons enter the C pore from the top.

Interestingly, in the predicted model, S5 is longer than other transmembrane segments and sticks out of the membrane on the extracellular side. The first 1/3 (N227-T235) of the putative L5–6 was predicted to be an extended part of the S5 helix, leaning toward the C domain (Fig. 7a). This is similar to what was seen in the cryo-EM structure of chicken Otop3^23^. The other 2/3 of this loop spins across the top of the N pore. Since the loop is missing in all three available cryo-EM structures^22,23^, it must be structurally flexible. Theoretically, it will be able to reach all three putative pores and regulate proton conductance. In the modeled structure, H229 stays at the beginning part of the extended helix of S5 and faces the C domain (Fig. 7a, b). In this sense, H229 may not actually stay on the flexible loop linking S5 and S6 as we were assuming. Spatially, H229 is not close to any putative proton-conducting pore in the predicted model. This is consistent with the SCAM results showing that H229C is not located directly in the proton-conducting pathway. In the model, H229 was predicted to interact with F580 in L11–12 through π–π stacking, and potentially with E575 through a hydrogen bond (Fig. 7b). If these interactions indeed exist in the structure of human Otop1, then there is a possibility that mutations of H229 alters these interactions and cause conformational changes in both L5–6 and L11–12, which may affect the proton gating of the Otop1 channel. In the model, R232 was predicted to point to the pore formed in the intrasubunit interface and the S7/S12 gap (Fig. 7b), suggesting a potential role of this residue in regulating proton permeation. Indeed, we noticed that the R232Q mutation leads to significant curvature of the *I*–*V* curve of Otop1 (Fig. 2e), suggesting that this mutation leads to a change in the voltage regulation of the proton permeation.

Three putative proton-conducting pores in the N domain, C domain, and the intrasubunit interface were proposed based on the cryo-EM structures of the Otop proteins^22,23^. It is unknown whether the three pores conduct protons separately or if all of them cooperate to compose a single proton-conducting pathway. Previously, single mutations in either of the three pores were shown to greatly inhibit or completely abolish the proton currents of the zebrafish or mouse Otop1 and Xenopus Otop3^22,23^. These results indicate that the three putative pores may all contribute to the same conducting pathway. However, one cannot rule out the possibility that these mutations bring about significant changes in Otop protein structures that affect the channel function. In this study, we found that the L11–12 mutation D570N leads to >90% current reduction of the human Otop1 channel (Fig. 5e, g). The cryo-EM structure of zebrafish Otop1 suggests that this aspartic acid is not directly involved in any interaction with surrounding residues^23^. Thus, it should not play a key role in maintaining the Otop1 structure, and the D570N mutation most likely will not cause a dramatic change in protein structure, nor will it affect the pores in the N domain and the intrasubunit interface. Thus, the fact that D570N still almost completely abolishes channel function strongly suggests that either the three conducting pathways in the three pores need to synergize in proton conductance or there is only one single conducting pathway, and all three pores contribute part of it in some way. If the latter is true, then results from both the D570 mutations and the SCAM results suggest that proton permeation in the human Otop1 channel may start at the top opening of the C pore, with D570 regulating the proton conductance via its location in the pathway (Fig. 7c).

## Methods

### DNA constructs

cDNA of human Otop1 (NCBI accession # BC130430.1) was cloned into a modified pGEMHE vector. An HA tag was fused to the C-terminus unless otherwise indicated. All full-length Otop1 single or multiple amino-acid mutants and loop deletion and loop replacement mutations were generated with the Q5 Site-Directed Mutagenesis kit (New England Biolabs). Deletion mutations L5–6∆, L6–7∆, and L10–11∆ have amino acid deletions from H229-N253, K295-D309, and K453-L530, respectively. The loop replacement mutation L5–6/3HA had its amino acid E228-T255 replaced with three copies of HA tags that are bracketed with two glycine residues on each side. The loop replacement mutation L11–12/HA has its residues R566-V577 replaced with one HA tag that is bracketed with two glycine residues on each side.

### Electrophysiology

Stage V–VI oocytes were harvested and prepared from adult female *Xenopus laevis* following standard procedures^33^. cDNA constructs were linearized and used for in vitro synthesis of RNA with T7 RNA polymerase. RNA (50 ng/oocyte) was injected into oocytes with a Drummond Scientific Nanoject III injector. Injected oocytes were incubated at 18 °C for 3 days before electrophysiology recording.

Otop1 channel currents were recorded with the two-electrode voltage clamp (TEVC) method. An OC-725D Oocyte clamp amplifier (Warner Instruments) with Digidata 1440A digitizer (Molecular Devices) and the pClamp 10 software (Molecular Devices) was used for recording. The standard bath solution contains 100 mM NaCl, 0.5 mM MgCl_2_, 5 mM HEPES, pH 7.5. For making the low pH solutions, 10 mM MES was used to replace HEPES in making the pH 6.0 solutions and 10 mM HomoPIPES was used for pH 5.0 and below. To test the acid-induced currents in other cations or anion solutions, 100 mM LiCl, CsCl, N-methyl-d-glucamine (NMDG^+^)–Cl, or 100 mM Na-gluconate was used to replace NaCl. The tetraethylammonium (TEA) solution contains 40 mM TEA methanesulfonate, 5 mM TEA chloride, 5 mM EGTA, and 100 mM HEPES or homoPIPES for pH 7.5 and 4, respectively.

Voltage protocols, including gap-free, voltage ramp, and voltage steps, were applied during TEVC recording. For gap-free recording, oocytes were clamped at −60 mV in the standard bath and low-pH bath solutions were applied for 20 s to invoke channel currents at different pHs. To obtain the current–voltage (*I*–*V*) relationship at a certain pH, oocytes were clamped at −60 mV and voltage ramps from −80 to +60 mV were applied to monitor channel current. When the current reaches its peak amplitude after applying acid solutions, 50 ms voltage steps from −100 to +100 mV were applied in 10 mV increments and the data were used to extract the *I*–*V* curves.

In the SCAM experiment, 1 mM MTSET, 1 mM MTSEA, 1 mM MTS-PtrEA, or 50 mM DTT was prepared by dissolving the chemical in bath solutions right before application. During the treatments, MTS reagents and DTT were applied for 2 and 1 min at pH 7.5, respectively. The effect of the MTS treatment was monitored by running either a gap-free recording at −60 mV during the treatment or a step protocol as described above before and after the treatment. The Baseline current before the pH 4 application was subtracted when measuring the amplitude of the currents at pH 4.

### Electrophysiology data analysis

Electrophysiology data were analyzed with both the Clampfit (Molecular Devices) and Excel (Microsoft) software. During data analysis, the leak current at pH 7.5 was subtracted from currents at the other pHs. For making the proton dose–response curves, the currents of every oocyte at other pHs were normalized to the current at pH 4 at −100 mV.

### Statistics and reproducibility

All TEVC recording results have been repeated with at least two batches of oocytes. The statistical significance among groups was calculated with the Student’s *t*-test, and *P* < 0.05 was considered statistically significant. Results ﻿are presented as means ± standard deviation (SD) in all bar graphs.

### Oocyte lysate preparation

In most cases, oocytes used in electrophysiology recording were collected to verify protein expression with western blot. To make the lysate, oocytes were first rinsed 3 times with a cold PBS solution. Then, the lysis buffer (10 µl/oocyte), which contains 1× PBS, 1% n-dodecyl-β-d-maltoside (DDM), protease inhibitor cocktail (Sigma-Aldrich), 1 mM EDTA, and 10% glycerol, was added onto the oocytes. Oocytes were homogenized by passing through a 25 G needle 10 times followed by sonication for 1 min. Samples were rotated at 4 °C for 1 h and vortexed for 5 s every 10 min. After centrifuging at 13,000 rpm for 30 min at 4 °C, the supernatant was then collected and treated with the SDS sample loading buffer at 37 °C for 30 min for SDS–PAGE.

### Surface biotinylation

Proteins on *Xenopus* oocyte plasma membrane were purified with the Cell Surface Protein Isolation Kit (Pierce) by following a modified protocol^34^. In brief, 3 days after RNA injection, oocytes (40 oocytes per sample group) were first washed twice with cold OR2 solution (82.4 mM NaCl, 2.5 mM KCl, 1 mM MgCl_2_, 10 mM HEPES, pH 7.6), then incubated with 0.4 mg/ml sulfo-NHS-SS-biotin in ice-cold OR2 for 30 min. The reaction was then quenched, and oocytes were washed following the manufacturer’s protocol. Oocytes were then homogenized and lysed as described above. Oocyte lysates were mixed with the NeutrAvidin beads at 4 °C overnight. The beads were washed, and proteins were eluted with 50 mM DTT. The eluted samples were treated and analyzed by SDS–PAGE and western blot.

### SDS–PAGE and Western blot

Cell lysate and surface biotinylated samples were run on 4–12% SDS–PAGE gels (Life Technologies) or homemade 10% SDS–PAGE gel and blotted with the mouse monoclonal anti-β-actin (GenScript, Cat. No. A00702, 1:1000 dilution) or rabbit monoclonal anti-HA (Cell Signaling, Cat. No. 3724T, 1:200 dilution) primary antibodies and IRDye 680-conjugated goat anti-mouse and IRDye 800CW-conjugated goat anti-rabbit secondary antibody (LI-COR Biosciences, Cat. No. 926-68070 and 926-32211, 1:10,000 dilution). Western blot images were scanned with the LI-COR Odyssey CLx imaging system.

### Animal use

All frog-related experimental protocols, complied with the NIH guideline, were approved by St. John’s University’s Institutional Animal Care and Use Committee (IACUC), and are reported according to the ARRIVE guidelines^35^. Fresh tricaine methanesulfonate (MS-222) solution (1 g/l, pH 7) was used for anesthesia, which is consistent with the AVMA guidelines.

### Reporting summary

Further information on research design is available in the Nature Research Reporting Summary linked to this article.

## Supplementary information

Supplementary information

Description of Additional Supplementary Files

Supplementary Data

Reporting Summary

## Data Availability

All data are contained within this article. Source data can be found in Supplementary Data. Uncropped blots are provided in Supplementary Fig. 8. All other raw data are available from the corresponding author on reasonable request.
