# Supplementary material for: The roles of two extracellular loops in proton sensing and permeation in human Otop1 proton channel
Source: Commun Biol. 2022 Oct 20;5:1110. doi: 10.1038/s42003-022-04085-2 (PMC9585144; doi:10.1038/s42003-022-04085-2)

Supplementary Information

The roles of two extracellular loops in proton sensing and permeation in human Otop1 proton channel

Bin Li[1,2], Yan Wang[1,2], Alexis Castro[1], Courtney Ng[1], Zhifei Wang[1], Haroon Chaudhry[1], Zainab Agbaje[1], Gabriella A. Ulloa[1], Yong Yu[1,*]

[1]Department of Biological Sciences, St. John's University, Queens, NY 11375, USA

[2]These authors contributed equally: Bin Li, Yan Wang

*** To whom correspondence should be addressed:**

Yong Yu

Department of Biological Sciences, St. John's University, St. Albert Hall 210, 8000

Utopia Parkway, Queens, NY 11439, USA

Phone: 1 (718)-990-1654; Email: yuy2@stjohns.edu

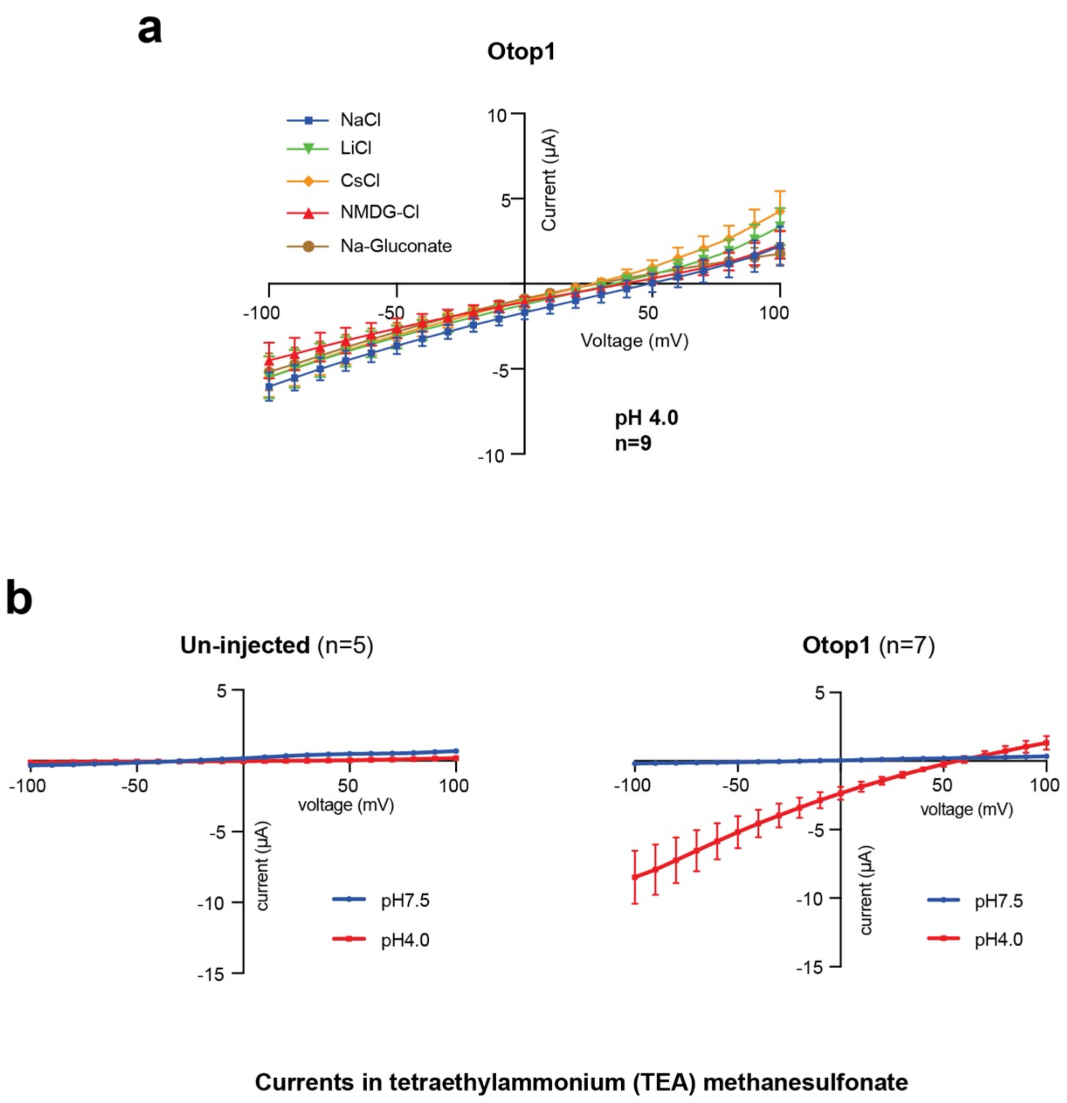

Supplementary Fig. 1 Acid-induced currents in Otop1-expressing oocytes were carried by protons conducted by the Otop1 channel. a Currents at pH 4 in bath solutions containing 100 mM of different cations (Na^+, Li^+, Cs^+, and $NMDG^+$) respectively, as well as in a bath solution containing 100 mM Na-Glutamate, without Cl^-. **b** Comparison of currents from un-injected oocytes and Otop1-expressing oocytes in pH 7.5 and pH 4. Bath solution contains 40 mM tetraethylammonium (TEA) methanesulfonate, 5 mM TEA chloride, 5 mM EGTA, and 100 mM HEPES or homoPIPES for pH 7.5 and 4, respectively.

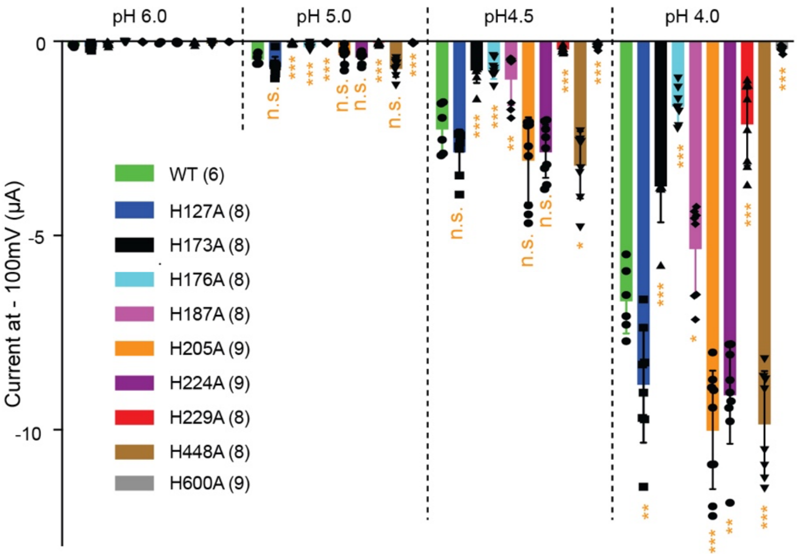

Supplementary Fig. 2 The effects of mutating histidine residues on channel activity of the human Otop1 channel. The scatter plot and bar graph showing the currents of the indicated WT and mutant Otop1 channels recorded at -100 mV at the indicated pHs. Data in the bar graphs are presented as mean ± SD. Currents of the mutants were compared to that of the WT with Student's t-test (*P < 0.05, **P < 0.01, ***P < 0.001, n.s.: no significance). Oocyte numbers for scatter plots and bar graphs are indicated in parentheses.

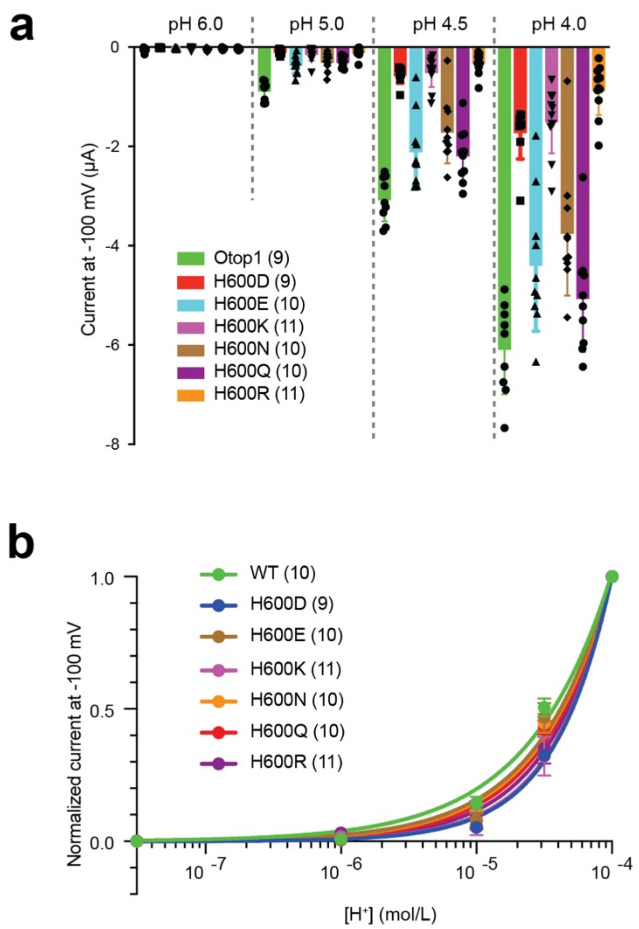

Supplementary Fig. 3 The pH sensing of Otop1 is not significantly changed by mutations of H600. a Scatter plot and bar graph showing the currents of the indicated channels recorded at -100 mV at the indicated pHs. Data in the bar graphs are presented as mean ± SD. Oocyte numbers for scatter plots and bar graphs are indicated in parentheses. **b** Proton dose-response curves showing the acid sensitivity comparison between the WT and the indicated mutant channels. The currents of every oocyte at other pHs were normalized to the currents at pH 4 at -100 mV. The data were fitted by nonlinear regression with variable slope.

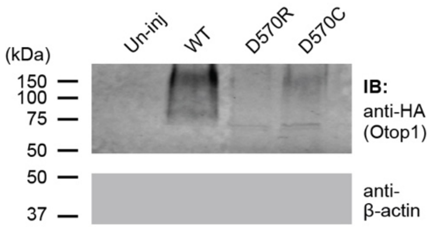

Supplementary Fig. 4 Western blot of the surface biotinylated samples shows a weak but clear expression of the Otop1-D570C mutants on the plasma membrane of *Xenopus* oocytes. The D570R mutant has a weaker surface expression compared to D570C. The surface samples are the same as the ones used in Fig. 5H but loaded with 1/3 more volume and blotted with a higher concentration of antibody.

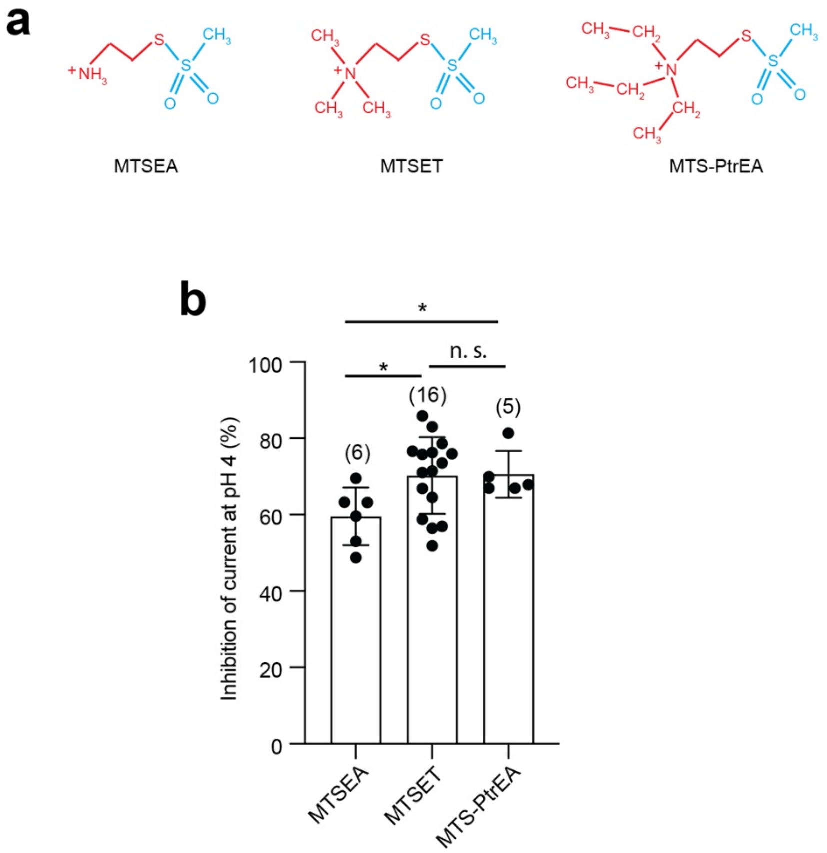

Supplementary Fig. 5 The inhibition effects of three MTS reagents on channel activity of the Otop1-D570C mutant. a Structures of the three MTS reagents used in our treatment. **b** The inhibition effects of indicated MTS reagents on channel activity of Otop1-D570C. Data were collected at pH 4.0 when oocytes were clamped at -100 mV. MTS reagents were applied at pH 7.5. Currents were compared with Student's t-test (*P < 0.05, n.s.: no significance). The numbers of oocyte numbers recorded in each group are indicated in parentheses.

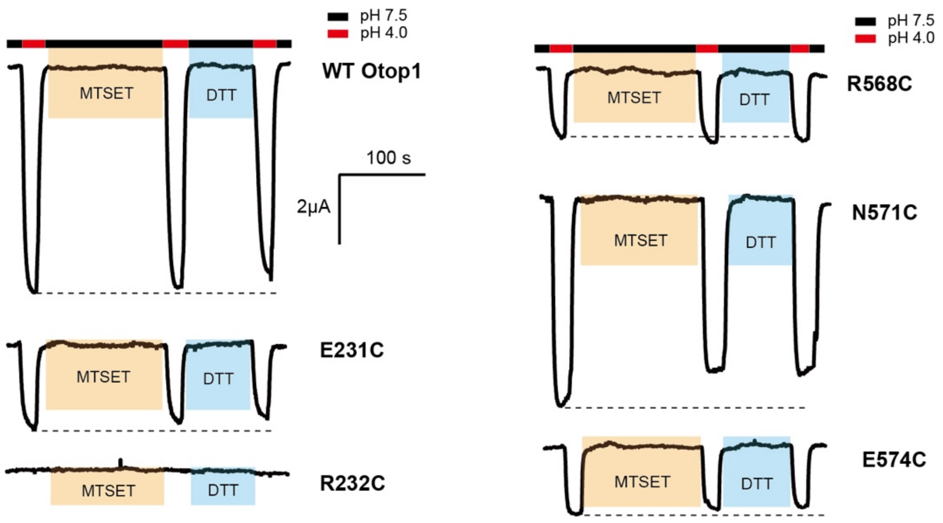

Supplementary Fig. 6 The effects of the MTSET and DTT treatment on the indicated WT and mutant human Otop1 channels. Gap-free recording showing the currents of WT and mutant Otop1 channels at pH 4 before and after 2 min treatment of 1 mM MTSET and 1 min treatment of 50 mM DTT. Oocytes were clamped at -60 mV. Dashed lines indicate the current sizes before MTSET treatment.

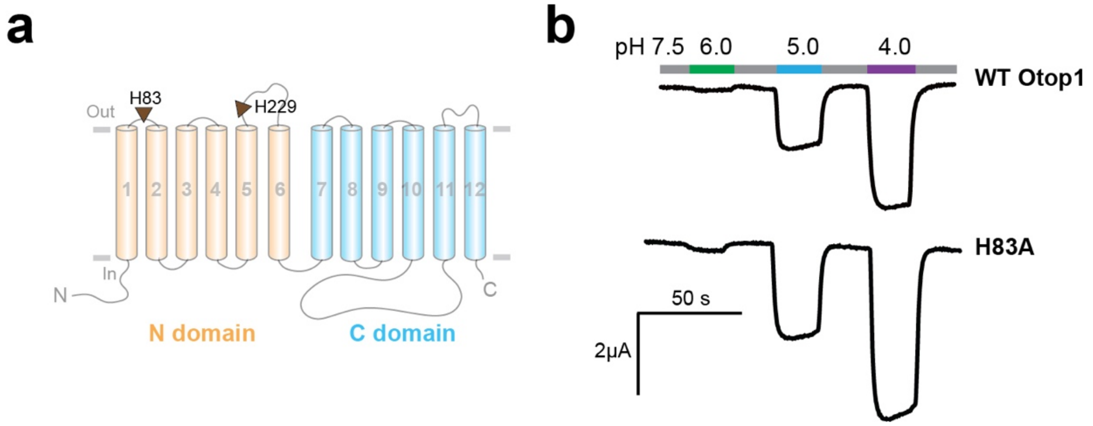

Supplementary Fig. 7 The H83A mutant has normal proton sensitivity. a Predicted positions of H83 and H229 in the topology structure of human Otop1. **b** Gap-free recording shows the currents of WT and H83A mutant at different pHs. Oocytes were clamped at -60 mV.

Supplementary Fig. 8 Uncropped western blots

Fig. 1f, anti-HA

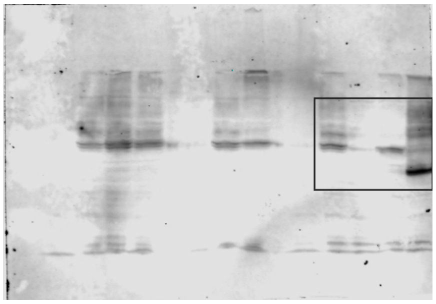

Fig. 1f, anti-actin

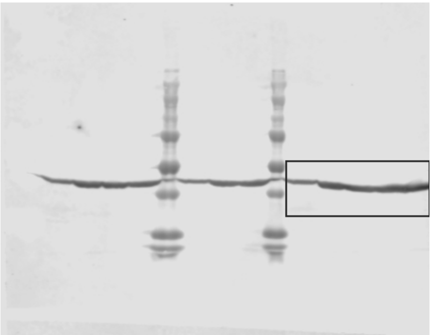

Fig. 1h, anti-HA

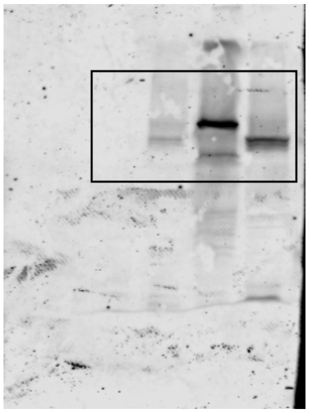

Fig. 1h, anti-actin

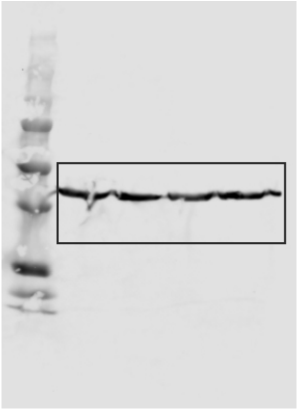

Fig. 2d, anti-HA

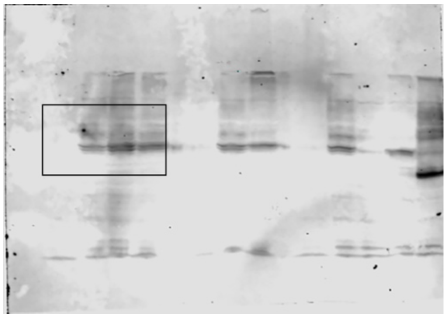

Fig. 2d, anti-actin

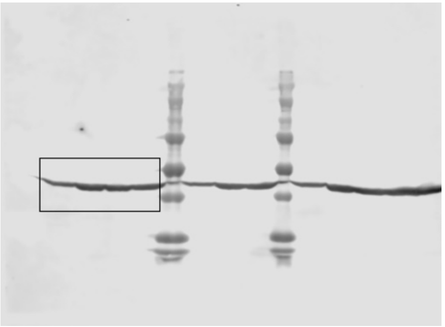

Fig. 2f, anti-HA

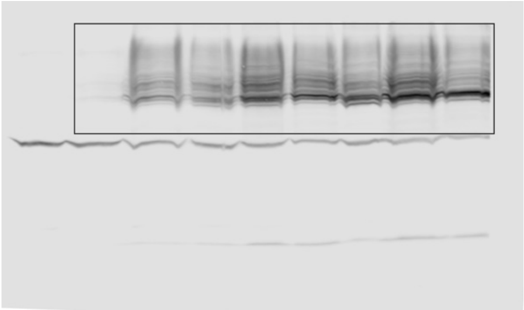

Fig. 2f, anti-actin

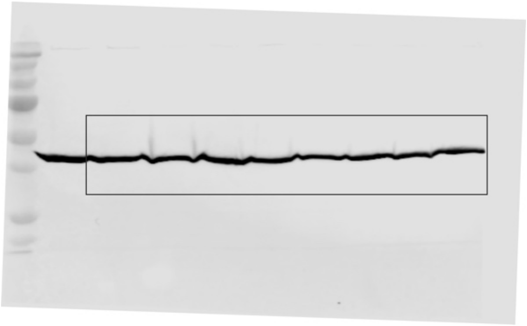

Fig. 2h, Lysates samples, anti-HA

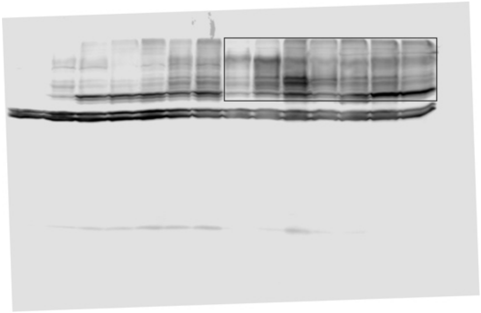

Fig. 2h, Surface samples, anti-HA

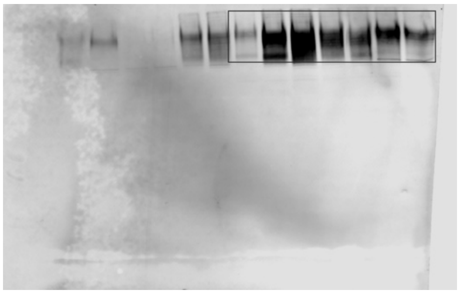

Fig. 2h, Lysates samples, anti-actin

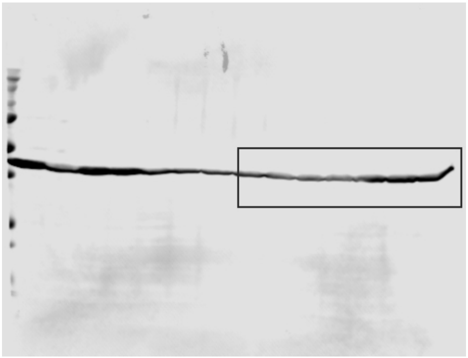

Fig. 2h, Surface samples, anti-actin

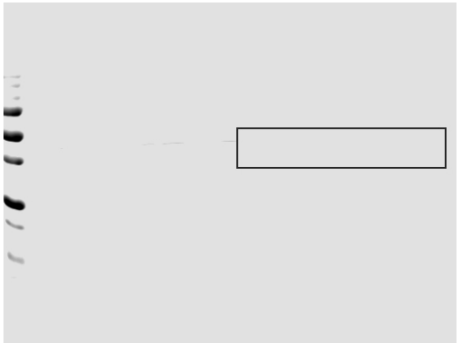

Fig. 4e, Surface samples, anti-HA

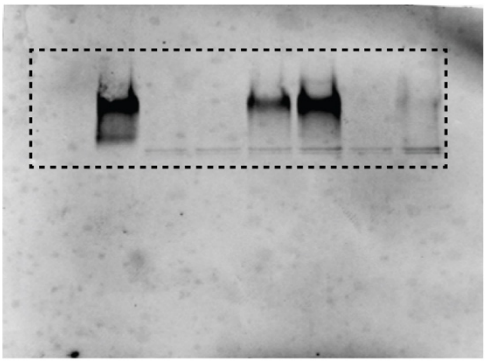

Fig. 4e, Surface samples, anti-actin

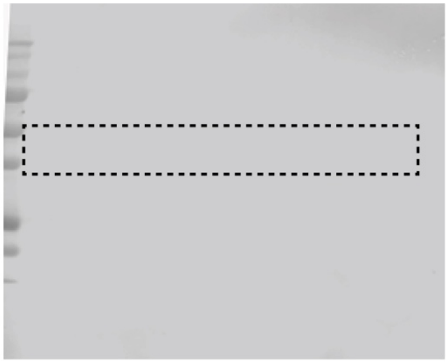

Fig. 4e, Lysate samples, anti-HA

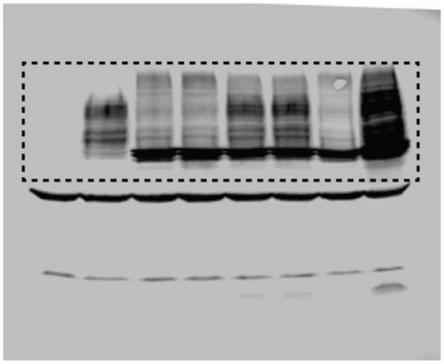

Fig. 4e, Lysate samples, anti-actin

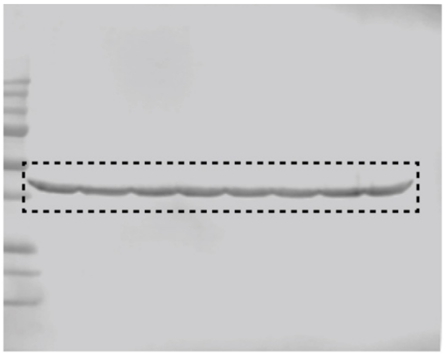

Fig. 5d, anti-HA

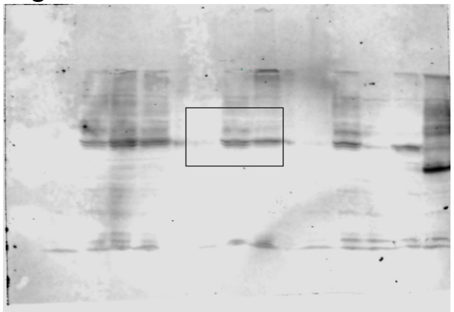

Fig. 5d, anti-actin

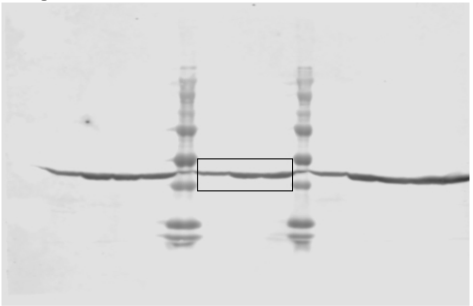

Fig. 5f, anti-HA

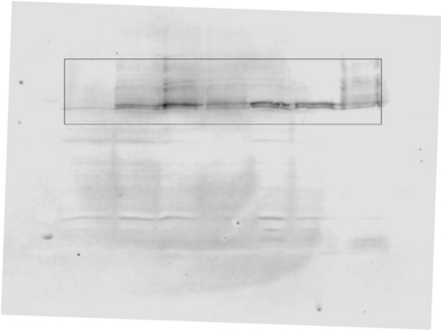

Fig. 5f, anti-actin

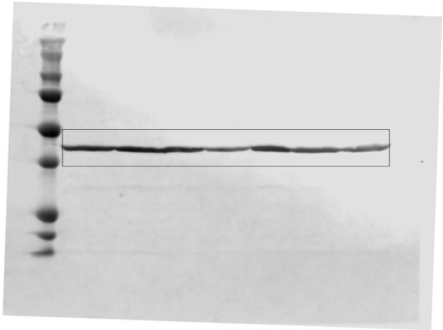

Fig. 5h, Lysate samples, anti-HA

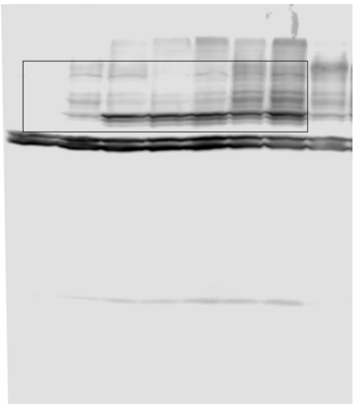

Fig. 5h, Lysate samples, anti-actin

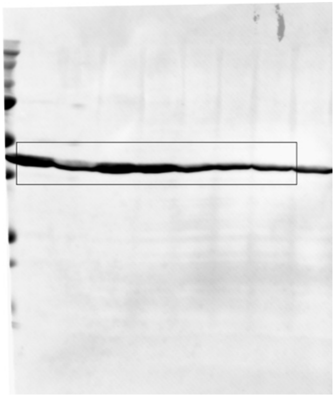

Fig. 5h, Surface samples, anti-HA

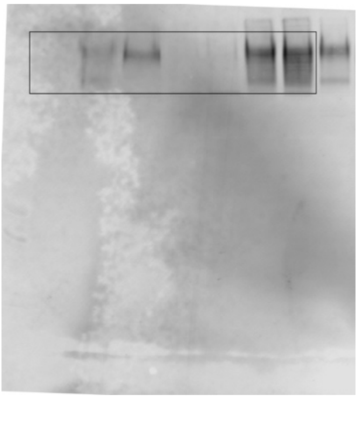

Fig. 5h, Surface samples, anti-actin

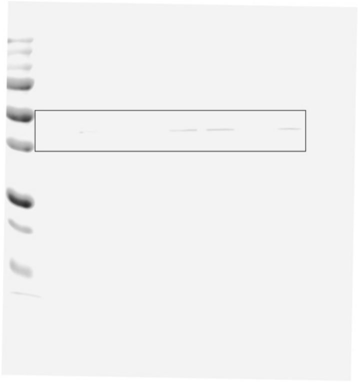

Supplementary Fig. 4, anti-HA

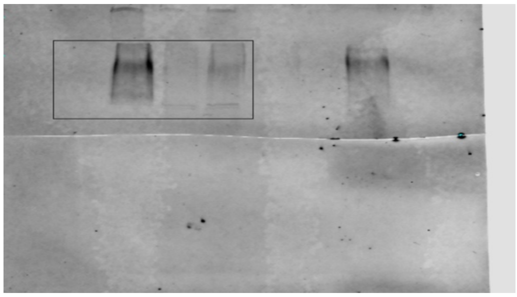

Supplementary Fig. 4, anti-actin

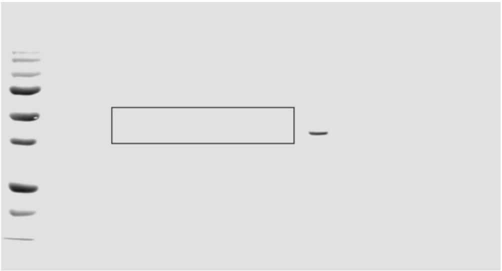

Supplement: Supplementary file 1 — Supplementary information [file 42003_2022_4085_MOESM1_ESM.pdf]
