# Supplementary material for: The roles of two extracellular loops in proton sensing and permeation in human Otop1 proton channel
Source: Commun Biol. 2022 Oct 20;5:1110. doi: 10.1038/s42003-022-04085-2 (PMC9585144; doi:10.1038/s42003-022-04085-2)
Supplement: Supplementary file 4 — Reporting Summary [file 42003_2022_4085_MOESM4_ESM.pdf]

nature portfolio

Corresponding author(s): Yong Yu
Last updated by author(s): Sep 28, 2022

Reporting Summary

Nature Portfolio wishes to improve the reproducibility of the work that we publish. This form provides structure for consistency and transparency in reporting. For further information on Nature Portfolio policies, see our Editorial Policies and the Editorial Policy Checklist.

Statistics

For all statistical analyses, confirm that the following items are present in the figure legend, table legend, main text, or Methods section.

n/a	Confirmed	
☐	☒	The exact sample size (*n*) for each experimental group/condition, given as a discrete number and unit of measurement
☐	☒	A statement on whether measurements were taken from distinct samples or whether the same sample was measured repeatedly
☐	☒	The statistical test(s) used AND whether they are one- or two-sided<br>*Only common tests should be described solely by name; describe more complex techniques in the Methods section.*
☒	☐	A description of all covariates tested
☒	☐	A description of any assumptions or corrections, such as tests of normality and adjustment for multiple comparisons
☐	☒	A full description of the statistical parameters including central tendency (e.g. means) or other basic estimates (e.g. regression coefficient) AND variation (e.g. standard deviation) or associated estimates of uncertainty (e.g. confidence intervals)
☐	☒	For null hypothesis testing, the test statistic (e.g. F, t, r) with confidence intervals, effect sizes, degrees of freedom and P value noted<br>*Give P values as exact values whenever suitable.*
☒	☐	For Bayesian analysis, information on the choice of priors and Markov chain Monte Carlo settings
☒	☐	For hierarchical and complex designs, identification of the appropriate level for tests and full reporting of outcomes
☒	☐	Estimates of effect sizes (e.g. Cohen's *d*, Pearson's *r*), indicating how they were calculated

Our web collection on statistics for biologists contains articles on many of the points above.

Software and code

Policy information about availability of computer code

Data collection	pClamp 10 (Molecular Devices) was used for electrophysiology recording
Data analysis	Clampfit 10 (Molecular Devices) was used for electrophysiology data analysis,Prism 8 (GraphPad) was used for data plotting, and PyMol-2.3.2 was used for structural figure preparation.

For manuscripts utilizing custom algorithms or software that are central to the research but not yet described in published literature, software must be made available to editors and reviewers. We strongly encourage code deposition in a community repository (e.g. GitHub). See the Nature Portfolio guidelines for submitting code & software for further information.

Data

Policy information about availability of data

All manuscripts must include a data availability statement. This statement should provide the following information, where applicable:
- Accession codes, unique identifiers, or web links for publicly available datasets
- A description of any restrictions on data availability
- For clinical datasets or third party data, please ensure that the statement adheres to our policy

Relevant data are available upon reasonable request.

Human research participants

Policy information about studies involving human research participants and Sex and Gender in Research.

Reporting on sex and gender	N/A
Population characteristics	N/A
Recruitment	N/A
Ethics oversight	N/A

Note that full information on the approval of the study protocol must also be provided in the manuscript.

Field-specific reporting

Please select the one below that is the best fit for your research. If you are not sure, read the appropriate sections before making your selection.

☒ Life sciences ☐ Behavioural & social sciences ☐ Ecological, evolutionary & environmental sciences

For a reference copy of the document with all sections, see nature.com/documents/nr-reporting-summary-flat.pdf

Life sciences study design

All studies must disclose on these points even when the disclosure is negative.

Sample size	In our electrophysiology recording, more than 5 oocytes (in most of cases more than 8) were tested for each mutants. The number is selected based on our previous experience and what people generally do in electrophysiology filed. This size is sufficient for performing the statistical test. Sample size was not applied to the structural analysis.
Data exclusions	Electrophysiology recording data from a small number of oocytes that have big leak currents (due to bad oocyte quality) were excluded.
Replication	All recording data have been successfully repeated with at least two to three batches of oocytes and all results were similar. Western blot was also repeated successfully.
Randomization	Xenopus oocytes were randomly selected for injection and current recording.
Blinding	Blinding was not performed in electrophysiology recording since the protocol was straightforward and was performed identically to the WT and mutants by computer controlled equipment. Data analysis was also performed by computer based method.

Reporting for specific materials, systems and methods

We require information from authors about some types of materials, experimental systems and methods used in many studies. Here, indicate whether each material, system or method listed is relevant to your study. If you are not sure if a list item applies to your research, read the appropriate section before selecting a response.

Materials & experimental systems

n/a	Involved in the study	
☐	☒	Antibodies
☒	☐	Eukaryotic cell lines
☒	☐	Palaeontology and archaeology
☐	☒	Animals and other organisms
☒	☐	Clinical data
☒	☐	Dual use research of concern

Methods

n/a	Involved in the study	
☒	☐	ChIP-seq
☒	☐	Flow cytometry
☒	☐	MRI-based neuroimaging

Antibodies

Antibodies used	Primary antibodies: mouse monoclonal anti-ß-actin (GenScript A00702-200), rabbit monoclonal anti-HA (Cell Signaling, 3724S). Secondary antibodies: IRDye® 680RD goat anti-mouse (LI-COR Biosciences, 926-68070) and IRDye® 800CW goat anti-rabbit IgG (LI-COR Biosciences,, 926-32211).

Validation	All antibodies are validated with negative control samples in our lab. The mouse anti-ß-actin antibody from GenScript and the rabbit monoclonal anti-HA antibody from Cell Signaling have been widely used in literature including previous publication from us.

Animals and other research organisms

Policy information about studies involving animals; ARRIVE guidelines recommended for reporting animal research, and Sex and Gender in Research

Laboratory animals	Oocytes from female Xenopus Laevis frog were used for expressing our ion channel and conducting electrophysiology recording.
Wild animals	The study did not involve wild animals.
Reporting on sex	N/A. Only oocytes were used.
Field-collected samples	The study did not involve samples collected from field.
Ethics oversight	Institutional Animal Care and Use Committee (IACUC) at St. John's University has approved the frog use protocol.

Note that full information on the approval of the study protocol must also be provided in the manuscript.
